# Study on the Compositional Analysis, Extraction Process, and Hemostatic and Anti-Inflammatory Activities of *Cirsium japonicum Fisch. ex DC.*–*Cirsium setosum* (Willd.) *MB* Extracts

**DOI:** 10.3390/molecules29091918

**Published:** 2024-04-23

**Authors:** Fanyu Kong, Zhongxue Fang, Biyue Cui, Jinshuang Gao, Changhai Sun, Shuting Zhang

**Affiliations:** 1 College of Pharmacy, Jiamusi University, Jiamusi 154000, China; kfy0804kfy@163.com (F.K.); fzx1234000@163.com (Z.F.); cby593448203@163.com (B.C.); 218053062@stu.jmsu.edu.cn (J.G.); 2 School of Functional Food and Wine, Shenyang Pharmaceutical University, Shenyang 110016, China

**Keywords:** *Cirsium japonicum Fisch. ex DC.*, *Cirsium setosum* (Willd.) *MB*, Compositional Analysis, extraction process, hemostatic, anti-inflammatory

## Abstract

*Cirsium japonicum Fisch. ex DC.* (CF) and *Cirsium setosum* (Willd.) *MB* (CS) are commonly used clinically to stop bleeding and eliminate carbuncles. Still, CF is mainly used for treating inflammation, while CS favors hemostasis. Therefore, the present study used UHPLC-MS to analyze the main chemical constituents in CF-CS extract. We optimized the extraction process using single-factor experiments and response surface methodology. Afterward, the hemostatic and anti-inflammatory effects of CF-CS extract were investigated by determining the clotting time in vitro, the bleeding time of rabbit trauma, and the induction of rabbit inflammation using xylene and lipopolysaccharide. The study of hemostatic and anti-inflammatory effects showed that the CF-CS, CF, and CS extract groups could significantly shorten the coagulation time and bleeding time of rabbits compared with the blank group (*p* < 0.01); compared with the model group, it could dramatically inhibit xylene-induced ear swelling in rabbits and the content of TNF-α, IL-6, and IL-1β in the serum of rabbits (*p* < 0.01). The results showed that combined CF and CS synergistically increased efficacy. CF-CS solved the problem of the single hemostatic and anti-inflammatory efficacy of a single drug, which provided a new idea for the research and development of natural hemostatic and anti-inflammatory medicines.

## 1. Introduction

*Cirsium japonicum Fisch. ex DC.* (CF) and *Cirsium setosum* (Willd.) *MB.* (CS), wild, perennial herbs of the family Asteraceae, are widely distributed in China and Europe [1]. As traditional Chinese medicine, they have a long history of thousands of years and have been recorded in the monographs of the Materia Medica throughout the ages. These herbs are used in whole form as medicine, which can be used to treat blood in urine, vomiting blood, leakage, blood in stool, and bleeding from trauma and are commonly used in folk medicine to treat inflammatory diseases [2,3]. According to “Liu Shangyi’s Commonly Used Drug Pairs in Clinical Analysis and Application”, CF and CS are commonly used clinically to cool blood, stop bleeding, dissipate blood stasis, remove toxins, and eliminate carbuncles. Their combined use can play a complementary role in promoting the therapeutic effect [4]. CF and CS are plants that have integrated medicine, food, and health care functions and have significant potential for development and utilization.

So far, more than 100 compounds have been isolated from these two medicinal plants, mainly including flavonoids, terpenoids, phenylpropanoids, alkaloids, and other chemical components. Flavonoids are among the main active ingredients in CF and CS [2,3]. Some studies have shown that CF and CS have a variety of biological activities such as hemostatic, anti-inflammatory, antioxidant, anticancer, antimicrobial, antiviral, antidiabetic, and hepatoprotective activity [5,6]. A large number of studies have shown that flavonoids can produce hemostatic effects by reducing capillary permeability and promoting vascular wall contraction, and they can also exert stronger anti-inflammatory effects by reducing the release of cytokines and inflammatory mediators [7,8]. Medicinal plants have attracted increasing attention from researchers around the world because of their wide range of action, low toxicity, and few side effects.

However, fewer studies have been reported on CF and CS. Therefore, the main chemical constituents, extraction process, hemostatic and anti-inflammatory activities of CF and CS were preliminarily studied in this study. This study provides an experimental reference for the further study of CF and CS.

## 2. Results and Discussion

### 2.1. Chemical Composition Analysis of CF-CS

UHPLC-HRMS/MS technology combined with Compound Discoverer 3.2^TM^; Mass Frontier7.0^TM^ software; and mzVault, ChemSpider, and mzCloud databases were used to analyze the chemical constituents of CF-CS ethanol extract. The identification results were as shown in Table 1, and 51 main active ingredients were preliminarily identified, including 4 amino acids, 8 organic acids, 22 flavonoids, 7 phenylpropanoids, 4 alkaloids, 1 phenol, 1 terpenoid, 1 anthraquinone and 3 other compounds.

**Figure 1:**
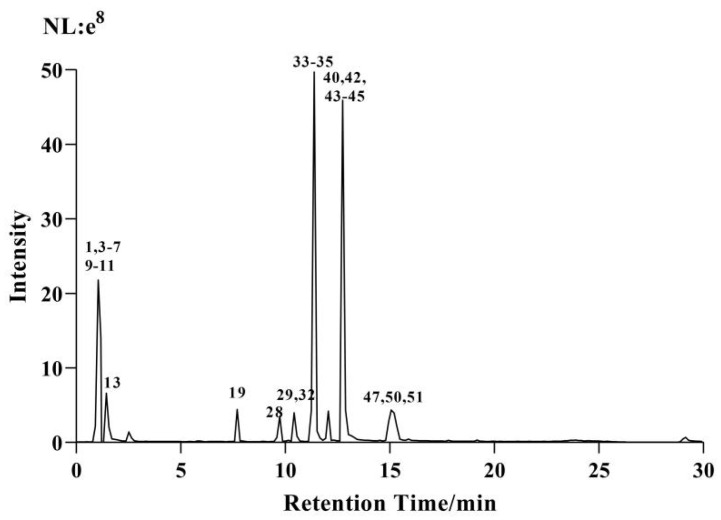
UHPLC–ESI–Q–Orbitrap base peak chromatogram (BPC) obtained in positive ion modes of CF–CS extract.

**Figure 2:**
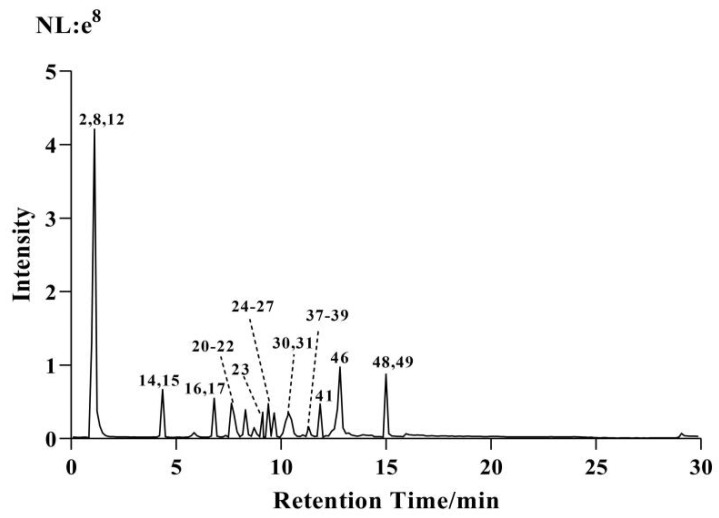
UHPLC–ESI–Q–Orbitrap base peak chromatogram (BPC) obtained in negative ion mode of CF–CS extract.

In the positive and negative ion scanning mode, the extracted ion chromatogram of the extract is shown in Figure 1 and Figure 2. The mass spectrometry information of 26 active ingredients was screened in the positive ion scanning mode, and 25 compounds were screened in the negative ion scanning mode.

### 2.2. The results of the Single-Factor Experiments

**Figure 3:**
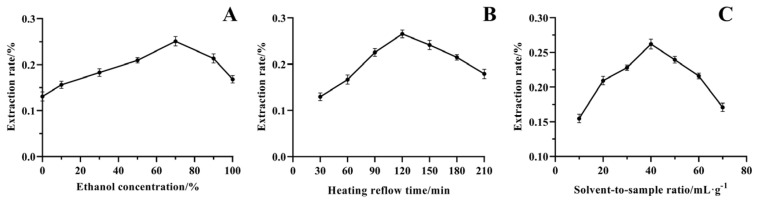
Effect of ethanol concentration ((**A**), %), reflux time ((**B**), min), and solvent-to-sample ratio ((**C**), mL·g^−1^) on the extraction rate of linarin.

As can be seen in Figure 3, the extraction of linarin from CF-CS increased with ethanol concentration (A). The highest extraction rate of linarin was achieved when the ethanol concentration was 70%, after which it started to decrease. The reason for this phenomenon may be that the polarity of 70% ethanol and buddleoside is relatively close. According to the principle of “similar compatibility”, the extraction rate is the highest at this time. However, with the increase in ethanol concentration, more organic solutes were dissolved, which led to a decrease in the extraction rate of buddleoside. Therefore, 50, 70, and 90% are used as the three levels of the Box–Behnken design.

The extraction rate of linarin gradually increased with the gradual increase in reflux time (B), reaching a maximum at an extraction time of 120 min. The reason may be that with the increase in time, buddleoside is continuously dissolved, and the maximum solubility is reached at about 120 min. As the reflux time continued to increase, some of the linarin was destroyed or oxidized, resulting in a gradual decrease in the extraction rate. Therefore, 90, 120, and 150 min were used as the three levels of the Box–Behnken design.

The extraction rate of linarin peaked when the solvent-to-sample ratio (C) was 40:1 (mL·g^−1^). This phenomenon may be due to the low solvent-to-sample ratio, the large viscosity of the overall system, and the poor fluidity of the medicinal materials in the solvent. The two failed to fully contact and the reaction was incomplete. Increasing the solvent-to-sample ratio, excessive solvent may consume energy, the energy absorbed by medicinal materials is reduced, and the extraction rate of buddleoside is also reduced. Therefore, 30:1, 40:1, and 50:1 mL·g^−1^ were used as the three levels of the Box–Behnken design.

### 2.3. Response Surface Experiment Results and Analysis

#### 2.3.1. The Results of the Response Surface Experiments

Based on the results of the single-factor experiments, a three-factor, the three-level analytical test was carried out to investigate the effects of ethanol volume fraction (A), reflux time (B), and solvent-to-sample ratio (C) on the extraction rate (Y) of linarin, using the principle of the Box–Behnken design, and the results are shown in Table 2.

#### 2.3.2. Model Fitting and Statistical Analysis

Multivariate regression was used to fit the above test results to obtain a quadratic polynomial regression equation, Y = 0.2657 + 0.0138A − 0.0188B − 0.0035C − 0.0014AB − 0.0075AC − 0.0082BC − 0.0594A^2^ − 0.0407B^2^ − 0.0093C^2^, which was subjected to a significant test and ANOVA, and the results are shown in Table 3.

As can be seen from Table 3, the regression model *p* < 0.0001 indicates that the quadratic regression equation model is significant; the misfit term *p* = 0.4583 > 0.05 suggests that the regression equation has a good fit; R^2^ = 0.9968 demonstrates that the model can adequately fit the experimental data; R^2^_Adj_ = 0.9926 indicates that the observed values correlate well with the predicted values; and the C.V.% = 1.71 suggests that the screening process of the model is accurate and reliable. The analysis of the variance of this model shows that among all the acting factors, the effect of the primary term C on the extraction rate of linarin was significant (*p* < 0.05). The impact of the preceding terms A and B; the interaction terms AC and BC; and the secondary terms A^2^, B^2^, and C^2^ were highly significant (*p* < 0.01). From the F-values in the table, it can be seen that the order of the effect of the factors on the extraction rate of linarin is reflux time (B) > ethanol concentration (A) > solvent-to-sample ratio (C).

#### 2.3.3. Graphical Interpretation and Optimization of Procedure

**Figure 4:**
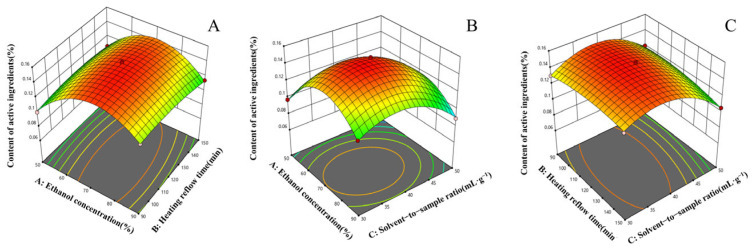
Response surface diagram of the effects of different factors on the yield of linarin. The variables of the two changes are ethanol concentration and reflux time (**A**); ethanol concentration and solvent−to−sample ratio (**B**); reflux time and solvent-to-sample ratio (**C**).

The response surface plots of the quadratic regression equation were obtained by using the software. Figure 4 depicts the response surface plots of the effect of the interaction between any two variables on the extraction rate of linarin. In the response surface plot, the steeper the surface plot, the more obvious the interaction between the variables. The interaction term (BC) had the most significant effect on the extraction rate of linarin, followed by (AC), and the interaction term (AB) was not significant, which was consistent with the analysis of variance in Table 3. The optimal extraction process was obtained as follows: ethanol concentration of 72.5651%, reflux time of 113.451 min, liquid-feed ratio of 38.5326:1 mL·g^−1^, and predicted content of 0.268891%. The optimized data were 70% ethanol concentration, 120 min reflux time, and 40:1 mL·g^−1^ for the convenience of the experiment. Three validation experiments were carried out to verify the correctness of the above scheme and the stability of the results, and the average linarin content was 0.2697%. The obtained experimental data were similar to the theoretical values, and the above results showed that the preferred extraction process of CF-CS was reasonable and feasible.

### 2.4. Validation Results of the Quantitative Method of Linarin

The range of linearity was established by injecting six different concentrations obtained by the dilution of a standard of linarin. Analytical curves for linarin were obtained considering the correlation between the peak area and the respective concentration of the standard. The standard curve equation obtained by the least squares method was y = 0.0340C − 0.2898, R^2^ = 0.9992, and the linear range was 21.60~129.60 μg·mL^−1^. As can be seen from the results, the linearity was satisfactory in all cases with correlation coefficients (R^2^ > 0.999). R values for the calibration curves higher than 0.99 verified that the linearity was adequate for the intended purpose.

Good precision, as revealed in the relative standard deviations (RSDs) for the peak area of linarin was 1.21%. 

For the stability test, the RSD of the peak area of linarin was 1.33%, indicating that the standard solution was stable for 24 h at ambient temperature.

The repeatability of the method was tested by the determination of a sample of CF-CS. The RSD for the contents of linarin was 1.43%.

The recovery test was conducted to evaluate the accuracy of this method. The recovery test of solution was obtained by adding a known amount of linarin standard solution to the six sample solutions. As shown in Table 4, the recovery rates for linarin were within the range of 98.2% and 102.5%. The RSD for the recovery rate was 1.64%.

### 2.5. Results of Linarin Content Determination

According to the results in Table 5, the average content of linarin in the alcoholic extract of CF-CS was 2.92 mg·g^−1^ with an RSD% of 1.43%, which is an accurate and reliable result. At the same time, the contents of linarin in CF, CS, and CF-CS were also compared, and the results showed that the contents of the three were similar.

### 2.6. Results of Coagulation and Hemostasis Tests

#### 2.6.1. Results of In Vitro Coagulation Tests

**Figure 5:**
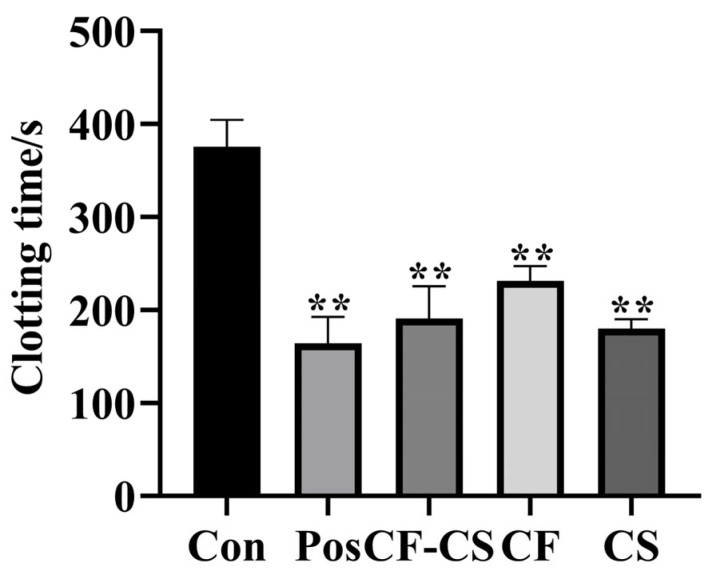
Effect of groups on clotting time in rabbits (x ± s, *n* = 6). Note: For each administration group compared to the blank control group, ** *p* < 0.01.

The results are shown in Figure 5. Compared with the blank group, the positive control group, CF-CS, CF, and CS could significantly shorten the coagulation time (*p* < 0.01), indicating that the experimental results were statistically significant. The results showed that CF-CS, CF, and CS had the effect of promoting blood coagulation.

#### 2.6.2. Results of Experiments on Traumatic Hemorrhage in Rabbits

**Figure 6:**
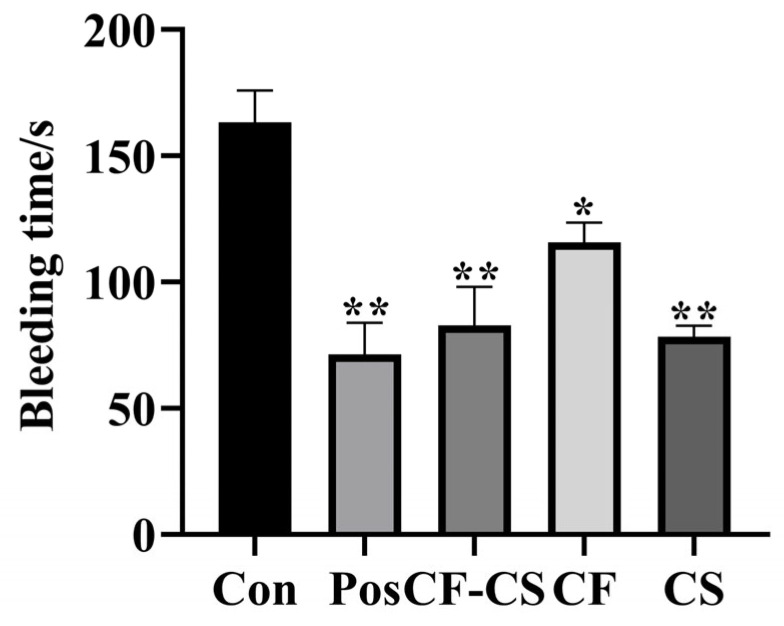
Effect of groups on bleeding time in rabbits. Note: For each administration group compared to the blank control group, * *p* < 0.05, ** *p* < 0.01.

Figure 6 displays the results. In comparison to the control group, the positive control group, CF-CS, and CS were shown to considerably reduce the bleeding time (*p* < 0.01), whereas CF was found to greatly reduce the bleeding time (*p* < 0.05). The experimental results demonstrate that CF-CS, CF, and CS have a distinct hemostatic effect.

### 2.7. Results of Anti-Inflammatory Experiments

#### 2.7.1. Experimental Results of Xylene-Induced Ear Swelling in Rabbits

**Figure 7:**
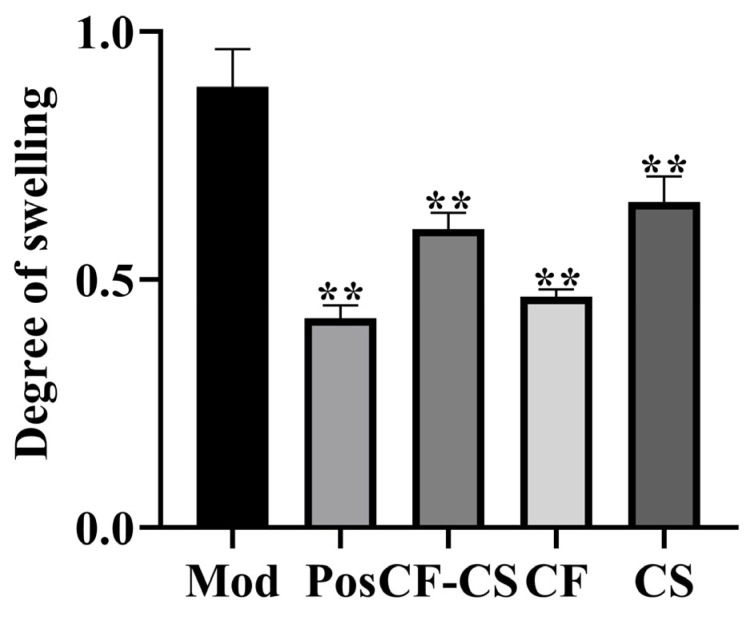
Effect of swelling in each administration group versus model control group. Note: For each administration group compared to the model control group, ** *p* < 0.01.

Figure 7 displays the results. In comparison to the model group, the positive control group, CF-CS, CF, and CS were able to considerably decrease the degree of swelling in rabbit ears (*p* < 0.01), indicating a statistically significant result. The findings demonstrated that CF-CS, CF, and CS were successful in suppressing xylene-induced edema in rabbit ears.

**Figure 8:**
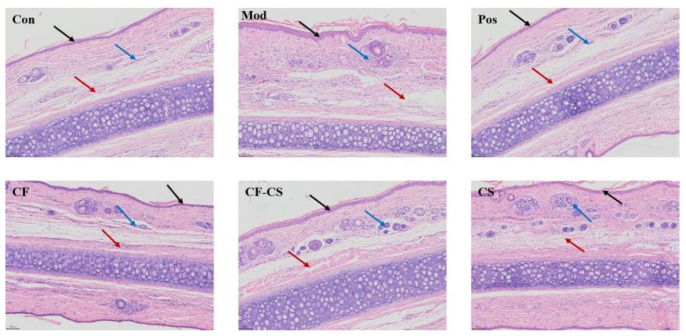
Histopathological observations of the ears of rabbits with swollen ears in each group (HE, ×200). Note: Thickening of the stratum spinosum (black arrow), disruption of the ear cartilage and subcutaneous connective tissue, widening of the interstitial space (red arrow), and infiltration of inflammatory cells (blue arrow).

Rabbit ear tissue sections were observed under a light microscope, as shown in Figure 8. Compared with the blank group, the thickening of the spinous layer in the model group, the destruction of the ear cartilage and subcutaneous connective tissue, the widening of the gap, and a large number of infiltration of inflammatory cells dominated by neutrophils could be seen in the surrounding area, which indicated that the modeling was successful; compared with the model group, the administration of the group could alleviate the extent of the thickening of the spinous layer, the degree of destruction of the ear cartilage and the subcutaneous connective tissue, and the infiltration of inflammatory cells. The results showed that all the administered groups had a protective effect and some anti-inflammatory effect on the ear tissues of rabbits with xylene-induced ear swelling.

#### 2.7.2. Results of the LPS-Induced Inflammation Experiment in Rabbits In Vivo

**Figure 9:**
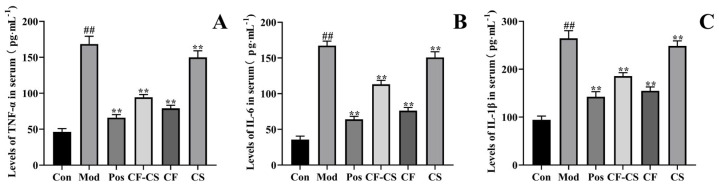
(**A**) The effect of the administered group on the serum level of TNF−α in rabbits; (**B**) the effect of the administered group on the serum level of IL−6 in rabbits; (**C**) the effect of the administered group on the serum level of IL−1β in rabbits. Note: Compared with the model control group in each administration group, ** *p* < 0.01; compared with the blank group, ^##^ *p* < 0.01.

To assess the suppressive impact of CF-CS, CF, and CS on inflammation in rabbits, ELISA was employed to measure the levels of TNF-α, IL-6, and IL-1β inflammatory cytokines in the rabbits’ serum. The findings from Figure 9 demonstrate that the levels of inflammatory factors TNF-α, IL-6, and IL-1β in the serum of rabbits in the model group were considerably elevated (*p* < 0.01) compared to the blank group. This provides evidence of successful inflammatory modeling in rabbits. The levels of TNF-α, IL-6, and IL-1β were significantly reduced (*p* < 0.01) in both the negative control group and the administration group compared to the model group. This suggests that the experimental results were statistically significant. Every administration group has the ability to efficiently suppress the levels of TNF-α, IL-6, and IL-1β in the serum of rabbits, thereby inhibiting the inflammatory response generated by LPS in rabbits.

### 2.8. Signal Pathway Analysis of CF and CS

The primary hemostatic and anti-inflammatory components of CF and CS were identified as linarin, acacetin, quercetin, pectolinarin, and pectolinarigenin by the use of network pharmacology and component identification. The AKT1, JUN, FOS, CASP3, IL6, MAPK1, and NFKBIA main targets are responsible for the hemostatic and anti-inflammatory actions exerted by these components [29,30,31,32]. Molecular docking technology was employed to align the active components with the target molecules. The results of molecular docking demonstrated that the binding energies between the crucial active components and the core target proteins were below −5 kJ·moL^−1^. This suggests that the active components and target molecules can form stable and effective interactions, thereby confirming the validity of the mechanism analysis. The molecular mechanism behind the hemostatic and anti-inflammatory effects of CF and CS may be associated with the IL-17 signaling pathway, TNF signaling pathway, and AGE-RAGE signaling pathway in the context of diabetic problems. The IL-17 signaling pathway is a well-known mechanism involved in the inflammatory response. It plays a special role in regulating the expression of IL-6 during the transcription of inflammatory genes. Additionally, it induces the production of chemokines and facilitates the adhesion, migration, and invasion of inflammatory cytokines. It has a significant impact on multiple inflammatory conditions. Research has demonstrated that by controlling the IL-17 signaling pathway, it is possible to decrease the concentration of inflammatory substances in the bloodstream, rectify the irregularity in the duration of bleeding, enhance the quantity of platelets, and promote their ability to clump together, thus achieving the process of blood clotting. The TNF signaling pathway is the primary inflammatory route activated by TNF-α. Macrophages release a protein that is composed of tiny molecules. They are cytokines that are part of the acute phase response and have a role in the overall inflammatory response throughout the body. The pro-inflammatory effects of TNF and IL-6 are strongly associated with angiogenesis. These substances exert their effects on the cells that line blood vessels, causing harm to these cells or disrupting their function. This can result in problems with blood flow, injury to the blood vessels, and the formation of blood clots. These effects can lead to the blockage of blood flow in tumor tissues, as well as bleeding, oxygen deprivation, and tissue death. They have the ability to block this pathway and function as hemostatic agents. The activation of the AGE-RAGE signaling pathway is associated with neuronal damage, inflammatory response, oxidative stress, and other related factors. Research has demonstrated that traditional Chinese medicine has the ability to hinder the inflammatory channels that are triggered by the AGE-RAGE signaling cascade, therefore exerting an anti-inflammatory effect.

## 3. Materials and Methods

### 3.1. Instruments, Reagents, and Drugs

A Dionex Ultimate 3000 RSLC ultra-high-performance liquid chromatograph, a Hypersil GOLD aQ column (100 × 2.1 mm, 1.9 μm), a Thermo Scientific Q Exactive Series mass spectrometer, a HESI-II ion source, methanol (chromatographically pure), formic acid (chromatographically pure), and acetonitrile (chromatographically pure) were purchased from Thermo Fisher Scientific (Shanghai, China). A Synergy-HT multifunctional enzyme labeler was purchased from Bio-TEK (Shanghai, China). Ethanol (analytically pure) and xylene (analytically pure) were purchased from Tianjin Komeo Chemical Reagent Co. (Tianjin, China). Linarin (MUST-22051011) was purchased from Chengdu Manster Biotechnology Co. (Chengdu, China). Lipopolysaccharide (LPS) was purchased from Dalian Meilun Biotechnology Co. (Dalian, China). Relevant inflammatory factor kits (TNF-α, IL-6, and IL-1β) were purchased from Wuhan Doctor Bioengineering Co. (Wuhan, China). Vanguard Red Ointment and Dexamethasone Hydrochloride were purchased from Golden Sky Heart Pharmacy (Jiamusi, China). CF and CS were purchased from Nanjing Tongrentang (Jiamusi, China), and identified by Prof. Zong Ximing, School of Pharmacy, Jiamusi University, as the whole herb of *Cirsium japonicum Fisch. ex DC*. and *Cirsium setosum* (Willd.) *MB*. of Cirsium japonicum, family Asteraceae.

### 3.2. Samples and Processing

The CF and CS herbs were dried in the shade to remove residual moisture. The samples were pulverized with a high-speed pulverizer and sieved with 65 mesh. The samples were placed under cool and dry conditions at 20 °C, sealed, and stored away from light.

### 3.3. Preparation of Test Solution

The CF powder and CS powder were weighed to 10.0 g and placed in a round-bottomed flask, and 70% ethanol was added to 800 mL, immersed for 30 min, then heated and refluxed for extraction and cooled. The abovementioned solution was centrifuged (3000 r·min^−1^) for 15 min, then the upper layer of the clarified liquid was aspirated. The supernatant was filtered through a 0.22 μm membrane, and the renewed filtrate was taken as the test solution.

### 3.4. UHPLC-MS Detection Conditions

#### 3.4.1. Chromatographic Conditions

The chromatographic conditions were as follows: aqueous formic acid (0.1%, *v*/*v*) and acetonitrile formic acid (0.1%, *v*/*v*) were used as mobile phases A and B, respectively. The elution gradient program was as follows: 0–2 min, 5% B; 2–22 min, 5–100% B; 22–26 min, 100% B; 26–27 min, 100–5% B; 27–32 min, 5% B. The flow rate was 0.3 mL·min^−1^, and the injection volume was 10 μL.

#### 3.4.2. Mass Spectrometry Conditions

The parameters of the high-resolution mass spectrometry were set as follows: sheath gas pressure of 40 psi; auxiliary gas pressure of 20 psi; purge gas pressure of 10 psi; capillary voltage of 3 kV; and ion transfer tube temperature of 320 °C. The AUG gas heating temperature was 350 °C; the collision gas was nitrogen; the normalized collision energies were 20, 40, and 60 eV; and the RF lens amplitude field strength (s-lens) was 60. Combined with selecting a complete primary MS scan, it automatically triggers the secondary MS scan mode (full MS-DD MS^2^). The resolutions of the primary and secondary high-resolution mass spectrometers were 70,000 FWHM and 17,500 FWHM, respectively; the ion scanning range was m/z 50–1500; the cycle counting was 3; the isolation window was 1.5 m/z; and the dynamic exclusion time was 5 s. 

### 3.5. Analysis of Chemical Constituents in CF-CS Extracts

Ultra-high-performance liquid chromatography–tandem mass spectrometry analysis uses positive and negative ion modes to scan for data simultaneously. Based on the precise mass-to-charge ratio of the fragmented ions, the primary high-resolution mass spectrometry data information (MS^1^) and secondary high-resolution mass spectrometry data information (MS^2^) were analyzed and processed by utilizing the Compound Discoverer 3.2™, Mass Frontier 7.0™ software and the mzCloud database, mzVault database, and ChemSpider database. 

### 3.6. Experimental Design of the Extraction Process

#### 3.6.1. Single-Factor Experiments

The ethanol concentration (A) (0%, 10%, 30%, 50%, 70%, 90%, 100%) was examined at a solvent-to-sample ratio of 20 mL·g^−1^ and a reflux time of 120 min. The reflux time (B) (30, 60, 90, 120, 150, 180, 210 min) was examined at a solvent-to-sample ratio of 20 mL·g^−1^ and ethanol concentration of 70%. The solvent-to-sample ratio (C) (10, 20, 30, 40, 50, 60, 70 mL·g^−1^) at 70% ethanol concentration and 120 min reflux time was used to determine the effect of each factor on the extraction rate of linarin in CF-CS.

#### 3.6.2. Box–Behnken Design Optimization

Based on the results of the single-factor experiments, the Box–Behnken design optimization was designed. Linarin extraction rate (Y) was used as the response value and ethanol volume fraction (A), reflux time (B), and liquid/feed ratio (C) as the response factors to design a 3-factor, 3-level Box–Behnken design optimization to optimize the extraction process of CF-CS using Design Expert 12 software. The factors and levels of response surface methodology are shown in Table 6.

### 3.7. Validation of Quantitative Method for Linarin

According to the guidelines for analytical method validation in Pharmacopeia of People’s Republic of China (volume IV) (version 2020), the linearity regression curves for linarin were obtained by plotting the peak areas (y) against the concentrations (x) of linarin standard solution. The precision of the method was evaluated by the same standard solution six times with successive injections. The stability test was performed by analyzing the same sample solution at 0, 2, 4, 6, 8, 12, 18, and 24 h. The repeatability was determined by preparing six sample solutions independently and calculating the RSD of the contents. The recovery test was conducted to evaluate the accuracy of this method. The recovery test of a solution was performed by adding a known amount of linarin standard solution to the six sample solutions and calculating the recovery rate and the RSD.

### 3.8. Determination of Linarin Content

The test solution described in Section 3.7 and was taken and analyzed using the UHPLC-MS method under the detection conditions in Section 3.4.

### 3.9. Study on the Role of Coagulation and Hemostasis

#### 3.9.1. In Vitro Coagulation Assay in Rabbits

For each assay, 70% ethanol was taken as a blank control and Yunnan Baiyao extract as a positive control. CF extract, CS extract, and 1 mL of CF-CS extract were placed in a 5 mL test tube, and three drops of disodium citrate were added. After blood was taken from the vein at the edge of the ear of rabbits, 1 mL was added to the abovementioned test tubes, and then 25 μL of 0.2 mol·L^−1^ CaCl_2_ was added and mixed well. The process was timed, and the test tubes were tilted every 30 s until the blood clotted and no longer flowed. The timer was then stopped, the coagulation time was recorded, and the measurement was repeated six times.

#### 3.9.2. Bleeding Test of the Marginal Artery of the Ear in Rabbits

Rabbits were anesthetized with 10% chloral hydrate (2 mL·kg^−1^, intravenously). The dorsal side of the rabbit ear was debrided, tapped so that the ear veins were engorged, and then disinfected with 75% alcohol wipes. Then, a bleeding wound was created by making a 1 cm transverse cut with a scalpel blade in the central part of the outer side of the ear, including at least the arteries and veins (in the proximal third of the streets in the rabbit’s ear). The bleeding wound was first allowed to sit for 5 s to ensure normal bleeding. Then, the same quality of hemostatic material (Yunnan Baiyao, CF-CS) was pressed onto the bleeding wound, and after 10 s of pressure, the pressure was stopped. The clock was started, during which time the outflow of blood from the surface was gently absorbed with a filter paper until there was no more blood that the filter paper could drink. The time of hemostasis was recorded, and the measurement was repeated 6 times.

#### 3.9.3. Data Processing

SPSS was used to process the data, and an independent samples t-test was performed for coagulation time and bleeding time. *p* < 0.05 was considered as a statistically significant difference, and *p* < 0.01 was regarded as a highly statistically significant difference.

### 3.10. Study of Anti-Inflammatory Effects

#### 3.10.1. Xylene-Induced Ear Swelling Experiment in Rabbits

Thirty-six rabbits were selected and randomly divided into six groups: the blank group, the model group, the positive control group (0.500 g·kg^−1^ of Jingwanhong ointment), the CF-CS group (equivalent to the amount of raw CF-CS 1.802 g·kg^−1^), the CF group, and the CS group (the dosage administered was the same as in the CF-CS group). In the model group, only modeling was carried out without drug administration. Each group was coated with the corresponding drugs on the anterior and posterior surfaces of the rabbit’s right ear once/d for 7 consecutive days, and 100 μL of xylene was applied uniformly on the inner and outer surfaces of the auricle of the rabbit’s right ear 1 h after the last administration, except for the blank control group, and the left ear was the control. After 30 min, the rabbits were euthanized, both ears were cut off, and after overlapping the ears and aligning the edges of the ears, the ear pieces were punched off with a 6 mm diameter perforator and weighed sequentially. The degree of ear swelling was calculated according to Formula (1a) and the rate of ear swelling according to Formula (1b). The weighed ear pieces were quickly placed in 4% paraformaldehyde for fixation, then histopathological sections were made, and the histopathological sections were observed under a light microscope.

(1a) $$\mathrm{Degree}\;\mathrm{of}\;\mathrm{swelling}\;\left(mg\right)=\mathrm{right}\;\mathrm{earpiecequality}-\mathrm{left}\;\mathrm{earpiecequality}$$

(1b) $$\mathrm{Swelling}\;\mathrm{inhibition}\;\mathrm{rate}\%=\frac{\mathrm{mean}\;\mathrm{ear}\;\mathrm{swelling}\;\mathrm{in}\;\mathrm{the}\;\mathrm{model}\;\mathrm{group}-\mathrm{mean}\;\mathrm{ear}\;\mathrm{swelling}\;\mathrm{in}\;\mathrm{the}\;\mathrm{administered}\;\mathrm{group}}{\mathrm{mean}\;\mathrm{ear}\;\mathrm{swelling}\;\mathrm{in}\;\mathrm{the}\;\mathrm{model}\;\mathrm{group}}\times 100\%$$

#### 3.10.2. LPS-Induced Inflammation in Rabbits

The grouping of experimental animals was the same as in Section 3.10.1, and the model group involved only modeling without drug administration. The blank and model groups were gavaged with sodium carboxymethylcellulose, 20 mL at a time; the positive control group was gavaged with dexamethasone 5 mg·kg^−1^, 20 mL at a time; the CF-CS group (equivalent to the raw CF-CS dose of 1.802 g·kg^−1^) was gavaged with the alcoholic extract of CF-CS, 0.35 g·kg^−1^, 20 mL at a time; and the CF and CS groups were administered the same amount as the CF-CS group. The gavage was performed continuously for 7 d, once a day. After 1 h of drug administration on day 7, the remaining groups, except the blank group, were subjected to intraperitoneal injection of 1 mL of LPS at a concentration of 200 μg·kg^−1^ to induce an inflammatory model in rabbits. Blood was collected after 3 h to detect changes in TNF-α, IL-6, and IL-1β serum levels.

#### 3.10.3. Data Processing

SPSS was used to process the data, and an independent samples *t*-test was performed for TNF-α, IL-6, and IL-1β levels in serum. Statistically significant differences were defined as follows: *p* < 0.05 was considered significant, and *p* < 0.01 was considered highly significant.

## 4. Conclusions

This study aimed to evaluate the primary chemical components, extraction procedure, as well as the hemostatic and anti-inflammatory properties of the alcoholic extract of CF-CS. The primary active components of CF-CS extract were initially discovered using UHPLC-MS, in conjunction with mzVault, ChemSpider, and mzCloud databases, as well as the relevant literature. A total of 51 active constituents were detected, comprising 4 amino acids, 8 organic acids, 22 flavonoids, 7 phenylpropanoids, 4 alkaloids, 1 phenol, 1 terpenoid, 1 anthraquinone, and 3 other chemicals. Subsequently, the extraction rate of linarin, a constituent of the index, was evaluated. The extraction procedure of CF-CS alcohol extract was optimized using single-factor experiments and the response surface method. The ideal extraction parameters were determined to be as follows: an ethanol concentration of 70%, a reflux period of 120 min, and a liquid-to-feed ratio of 40:1 mL·g^−1^. Under these conditions, the linarin exhibited the greatest extraction rate of 0.2697%. Based on these criteria, the average linarin concentration was determined to be 2.93 mg·g^−1^. Furthermore, CF-CS extract demonstrated a notable ability to decrease clotting time and bleeding time in rabbits. It effectively mitigated xylene-induced ear swelling and reduced histopathological damage. Additionally, it exhibited inhibitory effects on the serum levels of inflammatory cytokines TNF-α, IL-6, and IL-1β in rabbits, thereby reducing the expression of inflammatory factors and improving the LPS-induced inflammatory response in rabbits. These findings suggest that they have the capacity to promote blood clotting and reduce inflammation.

## Figures and Tables

**Table 1:** Preliminary results of chemical constituents in the alcoholic extract of CF–CS.

No.	t_R_ (min)	Compound	[M+H]^+^ (m/z)	[M−H]^−^ (m/z)	Formula	Error (ppm)	MS^2^/m/z	Compound Class	Reference
1	0.91	DL-Arginine	175.11871		C_6_H_15_O_2_N_4_	−1.383	130.09734	amino acids	[9]
2	0.98	D-(-)-Quinic acid		191.0553	C_7_H_11_O_6_	1.494	173.04468, 155.03383, 137.02299, 127.03873	organic acids	[9]
3	1.05	Guanine	152.05647		C_5_H_6_ON_5_	−1.423	135.02994, 110.03501	alkaloids	[10]
4	1.05	DL-Stachydrine	144.10178		C_7_H_14_O_2_N	−0.869	102.05520, 98.09674	alkaloids	[11]
5	1.05	Trigonelline	138.05487		C_7_H_8_O_2_N	−0.616	121.06477, 110.06025, 94.06547	alkaloids	[12]
6	1.05	D-(+)-Proline	116.07080		C_5_H_10_O_2_N	1.679	98.06027, 70.06575	amino acids	[13]
7	1.05	Betaine	118.08638		C_5_H_12_O_2_N	1.057	100.07584, 72.08136	alkaloids	[9]
8	1.10	Malic acid		133.01288	C_4_H_5_O_5_	−2.028	71.01218, 115.00221	organic acids	[9]
9	1.17	4-Guanidinobutyric acid	146.09221		C_5_H_12_O_2_N_3_	−1.322	128.08156, 86.06042, 69.09171	organic acids	[14]
10	1.17	Salsolinol	180.10158		C_10_H_14_O_2_N	−1.806	163.07486, 151.07474, 137.05939	others	[15]
11	1.17	L-Phenylalanine	166.08600		C_9_H_12_O_2_N	−1.536	121.06471, 119.04895, 103.05429	amino acids	[9]
12	1.36	L-Pyroglutamic acid		128.03394	C_5_H_6_O_3_N	−2.184	82.02816	amino acids	[12]
13	1.43	Tyramine	138.09119		C_8_H_12_ON	−1.09	121.06479, 103.05445, 93.07022	others	[16]
14	4.35	Gentisic acid		153.01811	C_7_H_5_O_4_	−0.818	109.02798, 91.01743	organic acids	[17]
15	4.35	Protocatechuic acid		153.01811	C_7_H_5_O_4_	−0.818	109.02798, 91.02014	organic acids	[9]
16	6.81	Protocatechualdehyde		137.02312	C_7_H_5_O_3_	−1.464	119.01241, 123.00711	phenols	[12]
17	6.81	Salicylic acid		137.02312	C_7_H_5_O_3_	−1.829	119.01241, 109.02798	organic acids	[12]
18	7.63	Neochlorogenic acid		353.08817	C_16_H_17_O_9_	4.139	191.05528, 173.04469, 161.02319	phenylpropanoids	[9]
19	7.7	Chlorogenic acid	355.10144		C_16_H_19_O_9_	−2.587	179.05412, 191.05495, 173.04431, 161.02290, 135.04384	phenylpropanoids	[9]
20	8.03	Daphnetin		177.01843	C_9_H_5_O_4_	1.101	121.02822, 133.02815,	phenylpropanoids	[18]
21	8.03	Bergenin		327.07251	C_14_H_15_O_9_	1.085	312.03429, 234.02808, 192.02785	phenylpropanoids	[19]
22	8.30	Caffeic acid		179.03409	C_9_H_7_O_4_	1.144	135.04379, 117.03312, 107.04871	organic acids	[9]
23	9.12	3-O-Famprofazone		367.10391	C_17_H_19_O_9_	4.226	193.05006, 173.04462, 191.05490	organic acids	[9]
24	9.39	Rutin		609.14691	C_27_H_29_O_16_	3.117	301.03467, 285.04059, 271.02509, 227.03450, 151.00270	flavonoids	[9]
25	9.39	Kaempferol-7-O-neohesperidoside		593.15295	C_27_H_29_O_15_	2.962	577.97946, 285.04056	flavonoids	[20]
26	9.53	Hyperoside		463.08871	C_21_H_19_O_12_	3.472	316.02271, 301.03601, 287.02005, 271.02515	flavonoids	[9]
27	9.66	Luteolin-7-O-glucoside		447.09402	C_21_H_19_O_11_	4.098	285.04047, 151.00221, 133.02782	flavonoids	[21]
28	9.74	Scutellarin	463.08572		C_21_H_19_O_12_	−2.985	287.05402, 269.04480, 153.01784, 135.04370	flavonoids	[22]
29	10.42	Apigenin 7-O-glucuronide	447.09125		C_21_H_19_O_11_	−2.097	269.05927, 187.03818, 153.01785, 119.04910	flavonoids	[21]
30	10.49	4,5-Dicaffeoylquinic acid		515.12006	C_25_H_23_O_12_	3.218	353.08817, 191.05525, 179.03404, 173.04459, 135.04379	phenylpropanoids	[9]
31	10.49	3,5-Dicaffeoylquinic acid		515.11981	C_25_H_23_O_12_	0.603	353.08817, 179.03404, 173.04459	phenylpropanoids	[9]
32	10.56	6-O-Methylscutellarin	477.10190		C_22_H_21_O_12_	−1.787	299.06982, 284.04636, 272.11987, 186.01543, 168.00481, 137.05907, 121.02834	flavonoids	[22]
33	11.37	Pectolinarin	623.19562		C_29_H_35_O_15_	−2.289	477.13788, 315.08527, 300.06201	flavonoids	[2]
34	11.37	Linarin	593.18604		C_28_H_33_O_14_	−0.745	447.12650, 285.07465, 270.05136, 242.05632, 153.01770	flavonoids	[2]
35	11.79	Isorhamnetin	317.06442		C_16_H_13_O_7_	−3.656	168.00479, 153.01802	flavonoids	[17]
36	11.79	Fisetin	287.05423		C_15_H_11_O_6_	−2.733	269.04388, 165.04852, 157.05414, 153.01796, 135.04391	flavonoids	[23]
37	11.85	Nepetin		315.05148	C_16_H_11_O_7_	4.923	300.02765, 243.02972, 228.04236, 201.01862, 188.04700, 165.98959, 136.98665	flavonoids	[17]
38	11.85	Luteolin		285.04065	C_15_H_9_O_6_	4.51	241.05040, 171.05032, 153.02318, 135.02812	flavonoids	[17]
39	11.85	Quercetin		301.03561	C_15_H_9_O_7_	4.421	201.03961, 153.00240, 137.03880, 121.02810	flavonoids	[23]
40	12.06	Dihydrocapsaicin	308.22092		C_18_H_30_O_3_N	−3.57	290.21048, 262.21564, 184.13004, 137.07539, 122.05996	others	[24]
41	12.54	Corchorifatty acid F		327.21802	C_18_H_31_O_5_	4.338	309.12856, 291.19626	terpenes	[25]
42	12.68	Tricin	329.06696		C_17_H_13_O_7_	4.196	313.04358, 300.01981, 272.02499, 161.02321	flavonoids	[26]
43	12.74	Hispidulin	301.06982		C_16_H_13_O_6_	−2.805	286.04633, 168.00494, 153.99683, 119.01024, 107.02839	flavonoids	[17]
44	12.74	Apigenin	271.05939		C_15_H_11_O_5_	−2.619	243.06456, 229.04869, 197.05914, 163.03847, 153.01791	flavonoids	[2]
45	12.74	Genistein	271.05936		C_15_H_11_O_5_	−2.73	253.04716, 225.05359, 197.05914, 137.02843, 153.01791	flavonoids	[27]
46	12.81	Diosmetin		299.05624	C_16_H_11_O_6_	4.098	284.03287, 164.01042, 136.98671	flavonoids	[2]
47	14.93	Physcion	285.07495		C_16_H_13_O_5_	−2.806	257.07962, 242.05663, 213.05420, 153.01784	anthraquinones	[28]
48	15.00	Glycitein		283.06137	C_16_H_11_O_5_	4.487	240.04251, 223.03918, 211.03937	flavonoids	[17]
49	15.00	Acacetin		283.06137	C_16_H_11_O_5_	4.487	268.03796, 240.04251, 151.00246	flavonoids	[2]
50	15.20	Pectolinarigenin	315.08514		C_17_H_15_O_6_	−3.728	300.06189, 257.04364, 154.99695, 135.04384	flavonoids	[2]
51	15.34	Scopoletin	193.04913		C_10_H_9_O_4_	−2.099	178.02556, 133.02820, 105.03364	phenylpropanoids	[17]

**Table 2:** Design and results of the response surface experiment.

No.	Ethanol Concentration (A)/%	Heating Reflow Time (B)/min	Solvent-to-Sample Ratio (C)/mL·g^−1^	Extraction Rate/%
1	−1	0	−1	0.1771
2	1	0	−1	0.2214
3	−1	0	1	0.1878
4	1	0	1	0.2020
5	−1	−1	0	0.1716
6	1	−1	0	0.2005
7	−1	1	0	0.1336
8	1	1	0	0.1568
9	0	−1	−1	0.2295
10	0	−1	1	0.2362
11	0	1	−1	0.2117
12	0	1	1	0.1855
13	0	0	0	0.2698
14	0	0	0	0.2651
15	0	0	0	0.2600
16	0	0	0	0.2668
17	0	0	0	0.2668

**Table 3:** Response surface experiment variance analysis table.

Source	Sum of Squares	df	Mean Square	F-Value	*p*-Value	Significant
Model	0.0288	9	0.0032	239.71	<0.0001	significant
A	0.0015	1	0.0015	114.40	<0.0001	
B	0.0028	1	0.0028	210.99	<0.0001	
C	0.0001	1	0.0001	7.44	0.0295	
AB	8.122 × 10^−6^	1	8.122 × 10 ^−6^	0.6077	0.4612	
AC	0.0002	1	0.0002	16.95	0.0045	
BC	0.0003	1	0.0003	20.25	0.0028	
A^2^	0.0148	1	0.0148	1110.12	<0.0001	
B^2^	0.0070	1	0.0070	522.16	<0.0001	
C^2^	0.0004	1	0.0004	27.03	0.0013	
Residual	0.0001	7	0.0000			
Lack of Fit	0.0000	3	0.0000	1.06	0.4583	not significant
Pure Error	0.0001	4	0.0000			
Cor Total	0.0289	16				
R^2^	0.9968					
Adjusted R^2^	0.9926					
Predicted R^2^	0.9742					
Adeq Precision	46.8088					

**Table 4:** The recovery test results of linarin.

Compound	Original (µg)	Added (µg)	Found (µg)	Recovery Yield (%)	RSD (%)
Linarin	100.3307	101.0880	201.0308	99.6	1.64
	97.2304		200.8688	102.5	
	98.4671		200.2837	100.7	
	100.7545		200.0487	98.2	
	97.7450		200.0459	101.2	
	97.1247		200.6140	102.4	

**Table 5:** Results of linarin content determination.

Compound	No.	Content (mg·g^−1^)	Average Content (mg·g^−1^)	RSD%
Linarin	1	2.98	2.92	1.43
	2	2.92		
	3	2.88		
	4	2.91		
	5	2.87		
	6	2.95		

**Table 6:** Factors and levels table of response surface methodology.

Level	Ethanol Concentration (A)/%	Heating Reflow Time (B)/min	Solvent-to-Sample Ratio (C)/mL·g^−1^
−1	50	90	30
0	70	120	40
1	90	150	50

## Data Availability

All data generated or analyzed during this study are included in this published article.
